# Stargardt’s Disease: Molecular Pathogenesis and Current Therapeutic Landscape

**DOI:** 10.3390/ijms26147006

**Published:** 2025-07-21

**Authors:** Kunal Dayma, Kalpana Rajanala, Arun Upadhyay

**Affiliations:** 1 Ocugen India, 5th Floor, AYDIV IT Park, Financial District, Nanakramguda, Hyderabad 500032, Telangana, India; kunal.dayma@ocugen.com; 2 Ocugen, 11 Great Valley Parkway, Malvern, PA 19355, USA

**Keywords:** Stargardt’s disease, macular degeneration, ABCA4, lipofuscin, complement system, gene therapy

## Abstract

Stargardt’s disease (STGD1) is an autosomal recessive juvenile macular degeneration caused by mutations in the *ABCA4* gene, impairing clearance of toxic retinoid byproducts in the retinal pigment epithelium (RPE). This leads to lipofuscin accumulation, oxidative stress, photoreceptor degeneration, and central vision loss. Over 1200 pathogenic/likely pathogenic *ABCA4* variants highlight the genetic heterogeneity of STGD1, which manifests as progressive central vision loss, with phenotype influenced by deep intronic variants, modifier genes, and environmental factors like light exposure. *ABCA4* variants also show variable penetrance and geographical prevalence. With no approved treatment, investigational therapies target different aspects of disease pathology. Small-molecule therapies target vitamin A dimerization (e.g., ALK-001), inhibit lipofuscin accumulation (e.g., soraprazan), or modulate the visual cycle (e.g., emixustat hydrochloride). Gene therapy trials explore ABCA4 supplementation including strategies like RNA exon editing (ACDN-01) and bioengineered ambient light-activated OPSIN. RORA gene therapy (Phase 2/3) addresses oxidative stress, inflammation, lipid metabolism, and complement system dysregulation. Trials like DRAGON (Phase 3, tinlarebant), STARLIGHT (phase 2, bioengineered OPSIN) show promise, but optimizing efficacy remains challenging. With the key problem of establishing genotype–phenotype correlations, the future of STGD1 therapy may rely on approaches targeting oxidative stress, lipid metabolism, inflammation, complement regulation, and genetic repair.

## 1. Introduction

Stargardt’s disease, first described by German ophthalmologist Karl Stargardt in 1909 [1], is a type of juvenile-onset macular degradation [2]. It typically presents in the first or second decade of life with progressive central vision loss and characteristic white flecks in the retinal pigment epithelium (RPE) that cover the macula, and their progressive degeneration [3,4]. Activities requiring sharp vision, like reading and identifying faces, are significantly impaired, while peripheral vision often remains mostly intact [4]. It is a heritable disorder and the *ABCA4* gene, expressed at high levels in the rod photoreceptors and encoding ATP-binding cassette (ABC) transporter, has been identified as the causal gene for Stargardt’s disease and shows an autosomal recessive inheritance [5,6]. Stargardt-like disease, Stargardt 3 and Stargardt 4, are retinopathies with similar phenotype but with autosomal dominant inheritance associated with variants in ELOV4 [7] and PROM1 [8] genes, respectively. In this review, we have considered the Stargardt 1 disease, and it has been referred to as Stargardt’s disease throughout.

Robust data on the prevalence of Stargardt’s disease is lacking. A genomic database study, published in 2020, estimated the genetic prevalence of inherited retinal diseases (IRDs). The study indicated that more than one-third of the world population consists of healthy carriers of one mutation that can cause an autosomal recessive IRD. Among the 5.5 million people expected to be affected by an autosomal recessive IRD based on their genotype, about 1.98 million (33%) would have Stargardt’s disease genotype [9]. This gives an estimated prevalence of 1 in 4000 for Stargardt’s disease genotype (including late-onset disease). However, regional variations in prevalence are expected. The prevalence of Stargardt’s disease was observed to be 1 in 16,000 in Israel for 2023 [10] and 1 in 22,000 in The Netherlands for 2018 [11]. In the European Union, the prevalence of Stargardt’s disease has been reported as 1 in 10,000 in 2019 [12].

A female specific predisposition has also been observed for overall patients [13], patients aged 10–19 years [11], and for patients having mild *ABCA4* allele [14] suggesting that sex may be considered as a disease-modifying variable.

Despite the quantum of research on Stargardt’s disease, particularly the extensive studies on the disease associated variants present in the *ABCA4* gene, an approved therapy for this disease is lacking and promising therapy remains elusive.

Herein, we describe the etiology, pathophysiology, and epidemiology of Stargardt’s disease and dwell on the current therapeutic landscape of the disease while taking a deeper look on the candidate drugs and therapies evaluated in clinical trials.

### 1.1. The Visual Cycle and ABCA4 Protein

The gene product of *ABCA4* gene, the ABCA4 protein, is the photoreceptor specific flippase known as ATP-binding cassette subfamily A member 4, and it is a crucial component in the retina, specifically in photoreceptor cells [15]. This protein belongs to the ATP-binding cassette (ABC) transporter superfamily, a vast group of proteins that harness ATP hydrolysis to transport different molecules across cellular membranes. The designation “A4” signifies that it is the fourth gene in the “A” subfamily within this superfamily [16].

**Figure 1:**
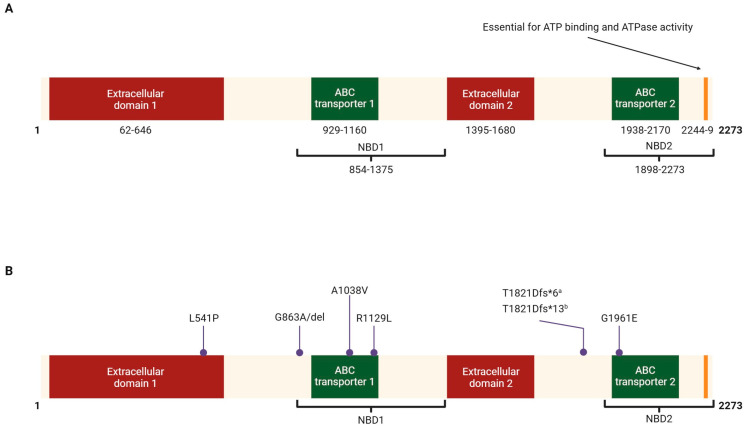
A schematic showing a linear structure of ABCA4 protein is depicted. (**A**) The domains of ABCA4 protein with amino acid numbers are shown. (**B**) ABCA4 variants that are present in at least 5% of patients are shown. ^a^ Frameshift results in a premature stop codon 6 amino acids downstream from the mutation site, ^b^ Frameshift results in a premature stop codon 13 amino acids downstream from the mutation site.

The ABCA4, a 2273 amino acid long protein, resides in the outer segments of photoreceptor cells, actively transporting vitamin A derivatives across the disc membranes of these segments following transduction of visual signal by photoreceptors [6]. ABCA4 protein has two nucleotide-binding domains (NBDs) and two extracellular domains (ECDs) [17]. The NBD-1 and NBD-2 each contain an ABC transporter region [18]. A terminal sequence essential for ATP binding and ATPase activity has also been described [19]. Various domains of ABCA4 have been shown in Figure 1A.

The human retina contains rods and cones photoreceptors, with rods highly sensitive to dim light and cones responsible for color vision and bright light detection [20]. In the rod and cone photoreceptors, the outer segment is a specialized cellular compartment responsible for detecting light and converting it into electrical signals through phototransduction. This segment contains a well-organized stack of disc membranes, which are densely packed with visual pigments rhodopsin or cone opsin [21,22]. Opsin, when covalently bound to 11-cis-retinal (a vitamin A derivative) via a Schiff base, is photoactive and referred to as rhodopsin in rods and cone opsin in cones. Upon photon absorption, 11-cis-retinal isomerizes to all-trans-retinal, triggering a series of conformational changes in opsin to its active state. The pigment is termed as “bleached” when it undergoes hydrolysis into opsin and all-trans-retinal and is regenerated when opsin recombines with 11-cis-retinal completing the visual cycle [23].

**Figure 2:**
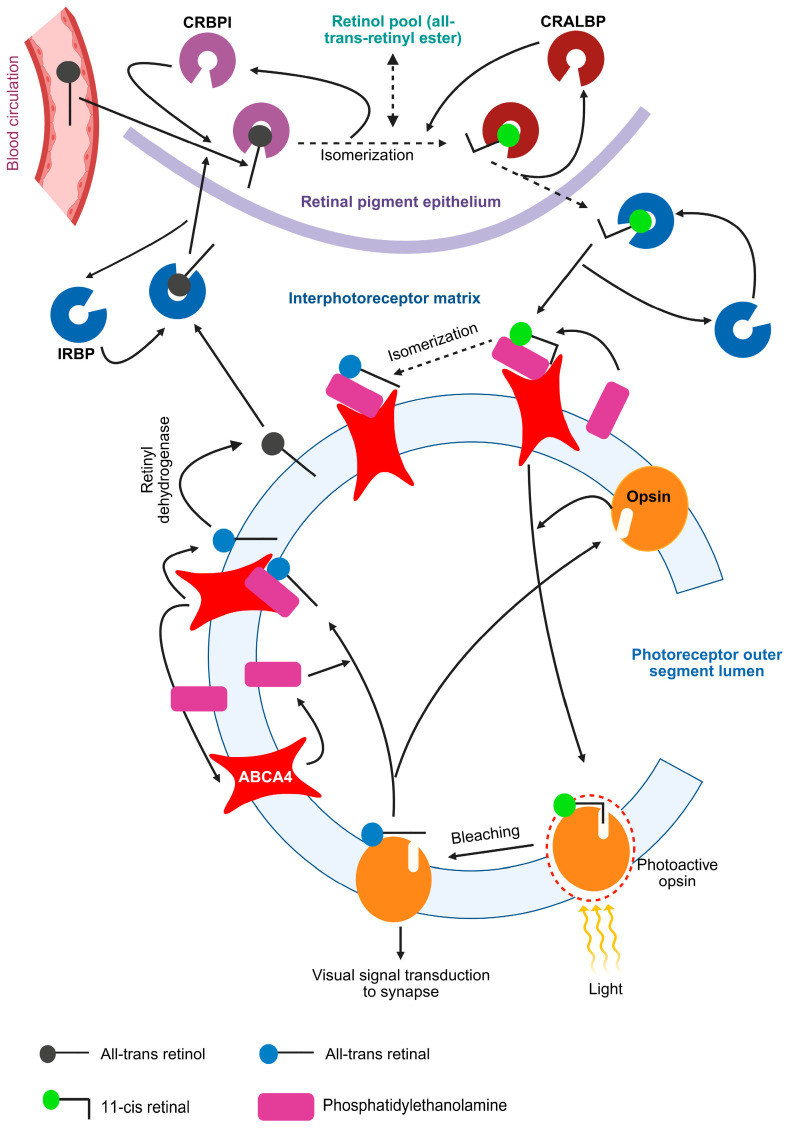
Visual cycle and ABCA4. The schematic depicts the steps of the visual cycle and involvement of ABCA4. Opsin binds to 11-cis-retinal and becomes photoactive (rhodopsin). The light, in visible range, induces the conformational change in the bound opsin bound 11-cis-retinal to convert it into all-trans-retinal which binds to phosphatidylethanolamine (PE) to form N-trans-retinylidene-phosphatidylethanolamine (NRPE). ABCA4 flips the NRPE to the cytoplasmic side from the lumen side of photoreceptor outer segment, where the all-trans-retinal is converted into all-trans-retinol by the action of retinyl dehydrogenase. The PE is recycled to the lumen side by the action of ABCA4. The all-trans-retinol binds to interphotoreceptor retinoid binding protein (IRBP), which takes it through the interphotoreceptor matrix to the retinal pigment epithelium cells. Inside the retinal pigment epithelium cells, the all-trans-retinal, which is sourced from photoreceptor outer segments and blood circulation, is bound to Cellular Retinol Binding Protein Type I (CRBPI). Series of steps lead to conversion of all-trans-retinol to 11-cis-retinol, which binds to Cellular Retinaldehyde Binding Protein (CRALBP) and is subsequently converted into 11-cis-retinol, which enters the interphotoreceptor matrix and binds to IRBP. In the retinal pigment epithelium cells, all-trans-retinol is also converted to all-trans-retinyl ester, which is a storage form of retinol. PE binds to the recycled 11-cis-retinal to form NRPE, which is flipped by the ABCA4 to the lumen side of the photoreceptor outer segment. After the flipping, 11-cis-retinal is available for binding to the opsin to start a fresh round of visual cycle.

ABCA4 localizes to the rim region and incisures of the retinal outer segment [24]. The all-trans-retinal, produced after the bleaching of rhodopsin, binds to phosphatidyl ethanolamine (PE) to form N-trans-retinylidene-phosphatidylethanolamine (NRPE) [25]. ABCA4 acts as a retinoid transporter moving NRPE, the Schiff-base adduct of retinal (all-trans-retinal) and phosphatidylethanolamine (PE), from the lumen to the cytoplasmic side of outer segment disc membranes of photoreceptors [25,26]. In the cytosolic side, all-trans retinal gets converted into all-trans-retinol by the action of retinyl dehydrogenase, which then binds to interphotoreceptor retinoid binding protein (IRBP), also known as retinol binding protein-3 (RBP3), and passes through the interphotoreceptor matrix to the retinal pigment epithelium cells [27]. Again, in the retinal pigment epithelium cells, all-trans-retinol undergoes a series of biochemical changes to eventually get converted into 11-cis-retinal [25,28]. Cellular Retinol Binding Protein (CRBP) binds the all-trans-retinol in retinal pigment epithelium cells. Subsequently, the conversion of all-trans-retinol to 11-cis-retinol occurs, which, in turn, binds to Cellular Retinaldehyde Binding Protein (CRALBP) and gets oxidized to 11-cis-retinal. The 11-cis-retinal moves to the interphotoreceptor matrix, binds to IRBP, and reaches the photoreceptor outer segment [27]. This recycled 11-cis-retinal binds to PE and enters the lumen of photoreceptor cells via ABCA4, making it available to create fresh rhodopsin and thus complete the visual cycle [28]. The retinal pigment epithelium cells also convert 11-cis retinol to all-trans-retinyl ester, which acts as a reservoir to store the retinol [29]. These events are depicted in Figure 2.

### 1.2. Etiology of Stargardt’s Disease

**Figure 3:**
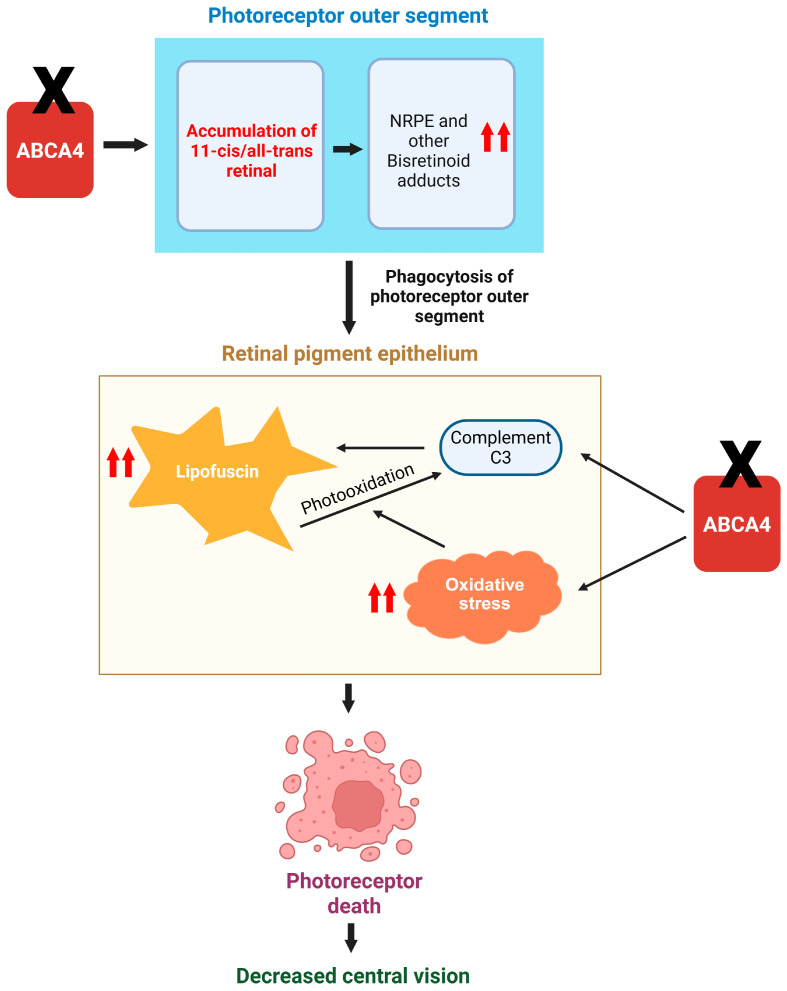
A schematic showing the general role of ABCA4 loss in Stargardt’s disease pathology. Loss of ABCA4 function results in accumulation of vitamin A derivatives in photoreceptors. Autophagy of photoreceptor outer membrane segments results in increased accumulation of lipofuscin. ABCA4 loss also results in increased complement C3 activation, which has been shown to increase lipofuscin accumulation. Also, photooxidation of lipofuscin, which gets augmented in the presence of increased oxidative stress due to loss of ABCA4, in turn activates the complement C3 in retinal pigment epithelium cells eventually leading to their cell death and decreased central vision.

Insufficiency of ABCA4, as observed in Stargardt’s disease, leads to the accumulation of NRPE in the outer segment of photoreceptors. After the outer segments are phagocytosed by retinal pigment epithelium cells, the NRPE is hydrolyzed to N-retinylidene-N-retinylethanolamine, a component of lipofuscin, and phosphatidic acid [30]. Bisretinoids, like NRPE, also undergo photooxidation followed by degradation [31]. Progressive buildup of lipofuscin in retinal pigment epithelium cells leads to degradation of the photoreceptors and subsequent loss in central vision (Figure 3). Vitamin A byproducts may also cause delayed dark adaptation by competing with vitamin A in binding to RPE65 and retinoic acid receptor [32].

As per a comprehensive study published in November 2023, 796 pathogenic, 452 likely pathogenic, and 971 *ABCA4* variants of uncertain significance were identified associated with Stargardt’s disease [33]. Another recent database study analyzing the *ABCA4* gene variants in about 11,000 patients having inheritable retinal disease identified around 1200 unique variants in the *ABCA4* gene that were pathogenic or likely pathogenic. Inheritance of Stargardt’s disease is autosomal recessive and compound heterozygous for Stargardt’s disease [34]. Pathogenic variants have also been observed on single allele in patients with Stargardt’s disease. Further analysis revealed deep intronic variants in some of these cases [35]. The variants in *ABCA4* gene are spread across the domains of ABCA4 protein [36]. In a German multicenter cohort study of 335 patients with Stargardt’s disease, 148 mutations in the *ABCA4* gene that are likely to impact disease pathology have been identified. About half of the patients (47.2%) had ≥2 mutations in the *ABCA4* gene, and 45.1% had mutations that were pathogenic or likely pathogenic. Single *ABCA4* mutation was observed in 28.4% of patients [37]. Similarly, in a study involving 133 Spanish patients with Stargardt’s disease, 48.1% reported ≥2 mutations and 24.8% reported single mutations in the *ABCA4* gene [38].

No mutational hotspots have been observed in the ABCA4 protein and mutations are observed in the protein across various functional domains. This underscores the complex heterogeneity in the correlation of genotype to phenotype in Stargardt’s disease. Table 1 and Figure 1B highlight the frequent mutations observed in ≥5% of patients with Stargardt’s disease across studies.

A recent study has demonstrated the effect of specific ABCA4 variants on disease progression. Square root-transformed atrophy growth, assessed by definitely decreased autofluorescence (DDAF), was slower in patients with the 5882G>A (0.0821 mm/year) and 4539+2001G>A (0.0686 mm/year) variants compared to those with 768G>T (0.1299 mm/year) and 5461-10T>C (0.1565 mm/year) [43]. In another recent study, the 5461-10T>C variant induced exon skipping in Stargardt’s disease patients and was found in compound heterozygous with G1961E which is a hypomorphic variant [44].

Various studies have reported deep intronic variants in patients with Stargardt’s disease with frequency ranging from 2% to 18% in the ABCA4 gene [36,37,45,46,47,48]. This highlights the potential of these variants in disease etiology, which is further underlined by the fact that these variants are not detected in the typical variant screening involving exons. A total of 353 deep intronic variants in ABCA4 gene have been identified in patients with Stargardt’s disease [49]. A multinational study involving 1054 probands with Stargardt’s disease and Stargardt’s like disease identified c.4253+43G>A (n = 100), c.4539+2001G>A (n = 64), and c.5196+1137G>A (n = 47) as the three most common deep intronic variants [49]. A recent study in a Chinese cohort of patients with Stargardt’s disease has shown that 45% of patients with no variants in exon region have deep intronic variants in the ABCA4 gene. Interestingly, the study also showed that such patients having deep intronic variants displayed a milder disease phenotype as compared to patients having biallelic variants in the exon region of the ABCA4 gene [50]. Deep intronic variants have also been reported to occur in trans with other variants occurring in a single allele [37]. The deep intronic variant c.769-784C>T has been found to be act as a modifier variant for G1961E variant, partly explaining the low penetrance of this variant [51] exemplified with the fact that its prevalence in normal population can be as high as 20% in west Africa [52]. The disease association of deep intronic variants warrants further studies with deep sequence analysis of larger number of patients.

Hypomorphic variants in ABCA4 have been associated with distinct disease phenotype. In a European cohort study in patients with Stargardt’s disease, the allele c.5603A>T (N1868I) was associated with majority (~80%) of late-onset cases and was determined as hypomorphic variant. The phenotype associated with this variant, late-onset disease with foveal sparring, was expressed only when this variant occurred in trans conformation with a deleterious mutation [53]. It is noteworthy that a deep intronic variant, c.4253+43G>A, has also been identified as an hypomorphic variant. This variant was detected in 2.6% of patients with Stargardt’s disease and was significantly enriched (14.4%) in patients having only one pathogenic allele. The variant, when present in trans with a loss-of-function ABCA4 variant, was associated with late symptom onset and foveal sparring Stargardt’s disease [54].

Mutations in Bestrophin-1 (*BEST1*) and Crumbs homolog 1 (*CRB1*) genes have also been reported in patients with Stargardt’s disease with wildtype *ABCA4* gene [55].

The root cause of Stargardt’s disease remains mutations in *ABCA4* gene, but the complete manifestation of disease pathology is likely to be affected by alteration in molecular pathways resulting from the lack of a functional ABCA4. This notion is supported by the fact that the Stargardt’s disease has juvenile onset. Data supporting this theory are shown in the preclinical studies described below.

#### 1.2.1. Role of Complement System

Dysregulation of the complement system has been observed in the outer retina of eyes of patients with Stargardt’s disease [56]. On the other hand, when the complement negative regulatory protein, complement receptor 1-like protein y (CRRY), is expressed in the retinal pigment epithelium cells using gene therapy in mice lacking ABCA4, there is a rescue of Stargardt’s disease phenotype. These mice expressing CRRY show a 2-fold reduction in bisretinoid levels [57]. Photooxidatin of NRPE, a component of lipofuscin, has also been shown to activate the complement C3 in in vitro studies [58,59]. Hence, Stargardt’s is associated with a dysregulation of the complement system on one hand, and checking the aberrant complement activation reduces the disease phenotype. These preclinical observations suggest a potential role of complement activation in the pathophysiology of Stargardt’s disease (Figure 3).

#### 1.2.2. Role of Oxidative Stress

As discussed above, retinal pigment epithelium cells phagocytose the outer segments of photoreceptors. An in vitro study involving cultured retinal pigment epithelium cells showed that the phagocytosis results in increased production of reactive oxygen species in the retinal pigment epithelium cells and the enzyme catalase protect these cells from the increased oxidative stress [60]. Reactive oxygen species are also sourced exogenously in retinal pigment epithelium cells due to exposure to visible light and high oxygen tension (70 mm Hg) [61]. The pigment melanin has been described as an antioxidant that protects retinal pigment epithelium cells from oxidative damage [62]. In mouse-based models of Stargardt’s disease, oxidative stress has been observed, which gets augmented in the absence of melanin. Mice lacking ABCA4 in the albino background showed increased oxidation of N-retinylidene-N-retinylethanolamine, which was checked by inhibition of rhodopsin regeneration. Although the levels of N-retinylidene-N-retinylethanolamine were not higher in ABCA knock out mice lacking melanin (albino) as compared ABCA4 knockout mice with melanin, the presence of increased oxidation of retinoid in albino mice indicates the role of light exposure in Stargardt’s pathology [63]. On similar lines, increased light exposure did not increase the lipofuscin formation per se but increased light-dependent oxidation of N-retinylidene-N-retinylethanolamine [64]. In the photoreceptors of albino mice lacking ABCA4, shortening of outer segment, disordering of disk membranes, damaging of inner segments, and morphological changes at the base of outer segments were observed. These changes were not observed in mice lacking ABCA4 but having intact melanin production [63]. These observations further highlight the potential role of increased oxidative stress in augmenting Stargardt’s disease pathology (Figure 3).

### 1.3. Stargardt’s Disease: Pathophysiology and Clinical Presentation

The macula is a small, round area located at the center of the retina, measuring about 5 mm in diameter. Despite its small size, it plays a crucial role in central vision, allowing you to focus on details like text, faces, and colors. The macula contains a high concentration of cone photoreceptors that are responsible for processing these details, while rods, found in the peripheral retina, aid with peripheral and night vision [65]. High expression of ABCA4 protein has been observed in the retina [66]. At the core of macula is the fovea, which is densely packed with cone photoreceptors and plays a vital role in tasks such as reading and facial recognition. In Stargardt’s disease, accumulation bisretinoid derivatives lead to lipofuscin accumulation in the fovea, which is particularly harmful due to the high density of photoreceptors and the region’s key role in detailed vision. Lipofuscin fluorescence has been observed to be up to three folds higher in patients with Stargardt’s disease as compared to healthy controls [51]. Lipofuscin buildup leads to the formation of hyperreflective debris on the apical side of retinal pigment epithelial cells seen as flecks. These flecks predominantly emit short-wave autofluorescence, and imaging studies have shown the associated loss of retinal pigment epithelium cells and corresponding thinning of outer nuclear layer in patients with Stargardt’s disease [67]. Consequently, the buildup of lipofuscin in the retinal pigment epithelium leads to damage to photoreceptors, causing the characteristic loss of central vision [68]. The clinical features of Stargardt disease involve progressive central vision loss, photophobia, and impaired color vision, often starting in childhood or adolescence. Fundus examination typically reveals characteristic yellow flecks and macular atrophy, while electrophysiological testing shows reduced ERG responses. The disease progresses slowly, with eventual total loss of central vision in severe cases. However, peripheral vision is often preserved for a long time, and visual prognosis can vary significantly based on disease progression and other factors [69]. With the gradual and progressive loss of central vision in Stargardt’s disease, a visual acuity ranging from 20/40 to 20/400 has been observed in patients [70].

As discussed earlier, numerous mutations in the ABCA4 gene have been associated with the Stargardt’ disease, highlighting the genetic heterogeneity which translates into the heterogeneity of disease onset and progression. Siblings carrying the same ABCA4 variant have been reported to display phenotypic discordance, showing substantial differences in disease progression including age at onset, visual acuity loss, and time to severe visual impairment [69,71].

The age of disease onset has been described as an important marker for disease severity. Early-onset Stargardt’s disease, occurring in first or second decade of life, has been described as a more severe disease with rapid decline of visual activity with progressive retinal degradation [72]. Milder disease phenotype has been observed with greater proportions in patients with late-onset disease (mean ages around 40 years). These patients were more likely to have hypomorphic alleles, while the severe phenotypes were associated with early-onset Stargardt’s disease (mean age around 17 years) [73]. Late-onset Stargardt’s disease has also been associated with foveal sparring. In a study involving 103 patients having Stargardt’s disease from The Netherlands with an age of onset of ≥45 years, about one-third of the patients displayed foveal sparring and a median 15.4 years passed from first diagnosis to foveal involvement [74]. Late-onset Stargardt’s disease can be misdiagnosed as age-related macular degeneration with one study reporting a misdiagnosis rate of 22% [11]. Phenotypic discordance, primarily affected by age and, as per some reports, by gender, highlights complex nature of genotype–phenotype relation of this disease.

### 1.4. Stargardt’s Disease: Diagnosis

Clinical features of Stargardt’s disease overlap with other retinopathies, making its careful diagnosis critical to avoid misdiagnosis. The diagnosis involves clinical examinations and imaging techniques described in brief below.

The clinical examination includes visual acuity testing and ophthalmoscopy.

- Visual acuity test: This test determines a patient’s ability to distinguish between two standard symbols or alphabets while viewing them from a standard distance (20 feet for US; 6 feet for UK). A visual acuity of x/y indicates that the patient can recognize symbols from a distance of x, whereas individuals with normal vision can identify the same symbols from a distance of y [75]. An age-related decline in the probability of maintaining a visual acuity of 20/40 has been observed in patients with Stargardt’s disease. A faster decline in visual acuity has also been reported in these patients after it dropped further from 20/40 and stabilized at 20/200 [76].
- Ophthalmoscopy: It is a clinical assessment to observe the internal structures of the eyes, specifically the fundus, of the patient through the dilated pupil using a ophthalmoscope [77]. The characteristic fundus appearance of Stargardt disease includes a macula with “beaten bronze” look, surrounded by distinct yellow flecks, either round or pisciform, located at the level of the retinal pigment epithelium [78].

Majorly, the key imaging techniques used in the diagnosis and prognostic monitoring of Stargardt’s disease are

- Fundus autofluorescence (FAF): This imaging technique detects the autofluorescence caused due to lipofuscin deposition in retinal pigment epithelium cells [79,80]. In the ProgStar study, which is a multinational, observational cohort study involving patients with Stargardt’s disease, around 250 patients each were analyzed retrospectively and prospectively with FAF as the primary outcome measure [81].
- Optical coherence tomography (OCT) and Spectral-domain OCT (SD-OCT): OCT uses light in the near-infrared region to examine the tissue. The delay in the reflected light provides a measure of the depth of reflection that determines its axial resolution. SD-OCT is an improved version of OCT with increased speed and image quality [82]. SD-OCT reveals outer retinal atrophy, including loss of the ellipsoid zone and thinning of the retinal pigment epithelium [83].
- Electroretinography (ERG): It can aid in the diagnosis and monitoring of Stargardt’s disease, but it is suggested to avoid these techniques as it involves bright light flashes that can exacerbate lipofuscin formation [84].

In 1976, Fishman classified Stargardt’s disease phenotypes on the basis of ophthalmoscopy [85]. Ophthalmoscopy imaging has the lacuna of not detecting the disease in early stages. In a study analyzing childhood-onset Stargardt’s disease, about one-fourth of patients showed no clinically detectable retinal lesions assessed by ophthalmoscopy at the time of presentation [72].

Three clinical phenotypes of Stargardt’s disease have been described based on FAF by Fujinami, et al., 2013 [86].

Type 1: A localized low FAF signal at the fovea, set against a uniform background, with or without perifoveal foci of increased or decreased signal.
Type 2: A localized low FAF signal at the macula, surrounded by a varied background, with widespread foci of increased or decreased FAF signal extending beyond the vascular arcades.
Type 3: Multiple areas of low FAF signal at the posterior pole, accompanied by a heterogeneous background and/or foci of increased or decreased signal.

These phenotypes differed in the rate of atrophy enlargement with disease severity increasing from type 1 to 3, highlighting the fact that patients who exhibit localized foveal atrophy tend to experience slower progression of central atrophy, whereas those with multiple atrophic lesions show a more rapid decline in central retinal structure over time [86].

FAF can be used for differential diagnosis of Stargardt’s disease along with multiple hereditary retinal disorders in children [87], Table 2.

### 1.5. Stargardt’s Disease: Therapeutic Landscape

No approved therapy is available for Stargardt’s disease at present. The clinical trial landscape for Stargardt’s disease is majorly dominated by small molecules followed by gene therapy and stem cell therapy. Trials that study small molecules, gene therapy, and stem cell therapy for Stargardt’s disease are listed in Table 3. To evaluate efficacy, the trials include structural assessments such as changes in lesion size, as well as functional assessments such as changes in visual acuity and retinal sensitivity.

#### 1.5.1. Small Molecules for Stargardt’s Disease

Tinlarebant is an orally administered retinal binding protein-4 (RBP4) antagonist [88]. RBP4 is responsible for delivery of retinol from liver to extrahepatic tissues including the eye [89]. The rationale behind using tinlarebant is to reduce the serum RBP-4, which would lead to a subsequent reduction in levels of retinol in the retina, thereby checking the levels of bisretinoids [88]. Tinlarebant has received a fast-track designation from the FDA in May 2022 [90] and received the Sakigake designation, which expedites the approval for innovative treatments targeting serious ailments, in Japan in June 2024. This designation offers prioritized consultation, pre-application support, expedited review, dedicated review partners, and extended re-examination periods for expediting the approval process [91]. The 2-year phase 2 trial for tinlarebant showed that patients receiving the drug showed sustained lower lesion growth as compared to patients enrolled in ProgStar trial having similar baseline characteristics (*p* = 0.001) [92]. The pivotal global phase 3 DRAGON trial has showed that tinlarebant is well-tolerated and has a consistent safety profile with stabilization of visual acuity in the 1-year interim analysis [93].

Similar to tinlarebant’s approach of reducing RBP-4 activity, STG-001 reduces the plasma levels of RBP-4, leading to reduced levels of vitamin A in retina [94]. It received an orphan drug designation in April 2019 from US FDA [95]. A phase 2a study comparing two doses of STG-001 and studying safety, tolerability, PK, and reduction in plasma RBP-4 levels was completed in April 2021 [96], Table 3. Subsequent trials for STG-001 have not been reported.

Metformin has been shown to reduce the levels of N-retinylidene-N-retinylethanolamine in the retina and retinal pigment epithelium/choroid of mice that were lacking ABCA4 gene [97]. A later study showed that metformin rescues the intra-cellular lipid accumulation by targeting lysosomal and fatty acid oxidation pathways in retinal pigment epithelium cells derived from induced pluripotent cells obtained from ABCA4 knockout mice [98]. The NEI is conducting a clinical trial to assess the safety and efficacy of oral metformin in slowing the progression of Stargardt disease. Participants aged 12 and older will take metformin for 24 months, with evaluations over at least 36 months [99].

Reducing the dimerization of vitamin A, which eventually leads to the formation of bisretinoids, is also a promising therapeutic approach. ALK001 is a vitamin A molecule with hydrogen at C20 position replaced by deuterium. It has shown reduced formation of vitamin A dimers in wildtype mice and rats [100]. A phase 2 trial assessing safety and tolerability of ALK-001 along with PK and lesion size and its extension trial (NCT4239625) assessing safety, tolerability, and PK is ongoing (Table 2).

Modulating the visual cycle to reduce the availability of vitamin A derivatives that subsequently form lipofuscin is another approach followed in the case of emixustat hydrochloride. This drug inhibits retinal pigment epithelium protein 65 (RPE65), which is responsible for an intermediate step in the molecular pathway converting all-trans retinal to 11-cis-retinal [101]. Emixustat received an orphan drug designation from the US FDA in January 2017 [102]. A phase 2 trial for emixustat, which was completed in 2017, tested its pharmacodynamics and safety in 23 patients with Stargardt’s disease [103]. This trail was followed by a phase 3 trial involving 194 patients with Stargardt’s disease which, upon completion in June 2022, showed a mean rate of change in macular atrophic area as 1.28 mm^2^/year in patients receiving oral emixustat (placebo: 1.31 mm^2^/year) at the end of 24 months [104]. A post hoc analysis of the same trial results including only the patients having smaller lesion size at baseline showed a 40.8% reduction in the progression of atrophic lesion with emixustat as compared to placebo (*p* = 0.02) [105]. No further trials have been reported for emixustat.

Soraprazan is a reversible inhibitor of H+/K+ ATPase. It has been shown to remove existing lipofuscin in monkeys [106,107]. A single intravitreal injection of soraprazan reduced lipofuscin in retinal pigment epithelium cells without toxicity. It also preserved photoreceptors in aged mice. The drug specifically accumulated in retinal pigment epithelium pigment granules, not in choroidal melanocytes, supporting its potential as a treatment [108]. Unlike ALK-001, which prevents additional vitamin A aggregates eventually leading to lipofuscin formation, soraprazan can also reduce the existing lipofuscin [106]. Soraprazan was given the orphan drug status by FDA in 2017 [109]. A double-blinded randomized phase 2 trial assessing lipofuscin by quantitative autofluorescence was started in 2019 in The Netherlands. As of the last update in April 2024, the trial status is still “recruiting” [110]. No further trials have been reported for soraprazan.

Dietary supplementation of omega-3 fatty acids and particularly DHA/EPA is another therapeutic approach that is being tried in clinical studies for Stargardt’s disease (Table 3). In mice lacking ABCA4, supplementation with omega-3 fatty acids adjusted to achieve the arachidonic acid to eicosapentaenoic acid ratio between 1 and 1.5, reduced the levels of N-retinylidene-N-retinylethanolamine approximately fourfold compared to mice not receiving the supplement (*p* < 0.05). Preservation of the outer nuclear layer (*p* < 0.01) and a reduction in the C3 complement system protein (*p* < 0.05) was also observed with omega-3 fatty acid supplementation [111]. The phase 2 trial testing omega-3 fatty acid supplementation in patients (N = 21) with Stargardt’s disease showed that, at 24 weeks of supplementation, there was an increase in best-corrected visual acuity (*p* = 0.003) and a higher score for perceived vision and subjective mood as compared to placebo [112].

As discussed above, complement system activation is a potential contributor to Stargardt’s disease etiology. A small-molecule inhibitor of complement C5 is being tested in a randomized phase 2b trial (Table 3). This global trial is active with 121 patients who would be given intravitreous injection of the study drug avacincaptad pegol [113].

#### 1.5.2. Gene Therapy for Stargardt’s Disease

Two kinds of gene therapy are under clinical trials for Stargardt’s disease, those supplementing wildtype ABCA4, and those aiming to modify the disease by expressing other molecules. Three phase 1/2 trials evaluating the safety and preliminary efficacy of gene therapy supplementing wildtype ABCA4 are ongoing.

ACDN-01 is an RNA exon editor delivered as a DNA construct via adeno-associated viral (AAV) vector. Once it is transcribed in the cells, ACDN-01 binds to the mutant ABCA4 pre-mRNA, via a highly specific binding domain, resulting in replacing the mutant RNA with the wildtype RNA [114]. This drug was given a fast-track designation by the FDA in January 2024 [115]. The organization developing the drug has claimed that ACDN-01 has shown durable and efficient RNA exon editing in human retinal explants and in primates [115]. A phase1/2 open-label dose ascending trial is ongoing to assess the safety and tolerability of this ACDN-01 delivered via a single subretinal injection [116].

Another AAV-based gene therapy, JWK006, which expresses *ABCA4* gene, is being assessed for safety and visual acuity in an ongoing phase 1/2 trial. It is administered via subretinal route [117].

A different gene therapy approach is followed in the case of virally carried ambient-light activatable multi-characteristic opsin (vMCO-010), also known as Sonpiretigene isteparvovec. It consists of an AAV-based expression of three opsins with separate activation peaks [118]. The protein product, multi-characteristic opsin, is a bioengineered fusion protein that is highly sensitive to light with broad spectrum responsiveness and faster kinetics to activate retinal cells in expressing the vMCO-001 in ambient light [119]. Outcomes at 24 weeks from the phase 2 trial, STARLIGHT, evaluating the safety and effectiveness of intravitreal injection of vMCO-010 in 6 patients with Stargardt’s disease, show favorable safety and tolerability. There was a gain of five points as per the Early Treatment Diabetic Retinopathy Study (ETDRS) chart in visual acuity and a three decibel gain in visual field in overall patients [119].

Another ABCA4 agnostic gene therapy approach is to express retinoic acid-related orphan receptor alpha (RORA), which is a nuclear hormone receptor. In the context of Stargardt’s disease, RORA regulates inflammatory response pathways that include the proteins CD59. It gets downregulated in Stargardt’s disease and is an inhibitor of the membrane attack complex (MAC) assembled with the help of complement C3 and C5, and ABCA4. RORA expression restores the expression of CD59 in mice lacking ABCA4 to levels that are comparable to wildtype mice [120]. As discussed above, dysregulation of a complement system has been implicated in Stargardt’s disease [56]. In mice lacking ABCA4, a rescue of Stargardt’s disease phenotype was observed in mice treated with AAV5 expressing human RORA (hRORA). Lipofuscin deposits, assessed by blue autofluorescence intensity, were significantly reduced upon RORA expression. The electroretinogram showed improvement in the scotopic b-wave amplitude and increased peak percentage recovery of baseline a-wave amplitude after photobleaching [120]. The role of oxidative stress in retina in augmenting Stargardt’s disease has also been discussed above. RORA protects from oxidative stress in cardiac fibroblast [121] and in cortical neurons [122]. A phase 1/2 study is being conducted to assess the safety and efficacy of OCU410, which uses an AAV5-based vector expressing hRORA, in patients with Stargardt’s disease. The trial is expected to be completed in October 2025 [123].

**Table 3:** Clinical trials for Stargardt’s disease using small molecules, gene therapy, and stem cell therapy.

Intervention	MOA	Outcomes Assessed	Phase	Sponsor	Reference
Small Molecules					
Tinlarebant	Retinol binding protein 4 antagonist	PK/PD, safety, change in atrophic lesion size	1b, 2/3	Mata Nathan	JPRN-jRCT2031240209 [124]
		Change in atrophic lesion size	3	Belite Bio, Inc.	NCT05244304 [125]
		Systemic and ocular safety and tolerability; the optimal dose for Phase 2	1/2	RBP4 Pty Ltd.	NCT05266014 [126]
STG-001	Reduces plasma levels of RBP4	Safety and tolerability	2	Stargazer Pharmaceuticals, Inc.	NCT04489511 [96]
Metformin	targeting lysosomal and fatty acid oxidation pathways in retinal pigment epithelium cells	Change in atrophic lesion size in ellipsoid zone band	1/2	National Eye Institute	NCT04545736 [99]
ALK001	Deuterated vitamin A; slows down the vitamin A dimer formation	Safety and tolerability, change in atrophic lesion size, PK	2	Alkeus Pharmaceuticals, Inc.	NCT02402660 [127]
		Safety and tolerability, PK			NCT04239625 [128]
Emixustat Hydrochloride	Inhibition of RPE65 and reducing 11 cis-retinal production	Change in retina electroretinogram; safety	2	Kubota Vision Inc.	NCT03033108 [103]
		Change in macular atrophic lesion	3	Kubota Vision Inc.	NCT03772665 [104]
Soraprazan	removal of lipofuscin in retinal pigment epithelium cells	Change in quantitative fundus autofluorescence	2	Katairo GmbH	NL-OMON48130 [110]
Omega-3 Fatty Acids Supplementation	Reducing complement C3 and accumulation of lipofuscin in retinal pigment epithelium cells	Visual acuity	N/A	Ophthalmos Research and Education Institute	NCT03297515 [129]
Zimura (Aptamer)	Complement C5 inhibition	Change in atrophic lesion size in ellipsoid zone	2	Astellas Pharma Global Development, Inc.	NCT03364153 [113]
Gene Therapies					
ACDN-01 RNA exon editor	AAV-based vector carrying a DNA construct encoding for an ABCA4 RNA exon editor	Safety and tolerability	1/2	Ascidian Therapeutics, Inc.	NCT06467344 [116]
JWK006	*ABCA4* gene expression via adeno-associated virus	Safety and visual acuity	1/2	West China Hospital	NCT06300476 [117]
OCU-410	RORA expression using Adeno-Associated Virus serotype 5	Safety and visual acuity	1/2	Ocugen	NCT05956626 [123]
SAR422459	Expression of ABCA4 using recombinant equine infectious anemia virus	Safety and tolerability	1/2	Sanofi	NCT01736592 [130]
vMCO-010	Viral expression of Opsin activated by ambient light	Safety, visual acuity, light-guided mobility, determination of shape and optical flow	2	Nanoscope Therapeutics Inc.	NCT05417126 [131]
Stem Cell Therapies					
autologous bone marrow-derived stem cells	Treatment of retinal and optic nerve damage	Safety and neuronal degradation in the eye	1	Pomeranian Medical University Szczecin	NCT03772938 [132]
		Visual acuity	N/A	MD Stem Cells	NCT01920867 [133]
					NCT03011541 [134]

#### 1.5.3. Stem Cell Therapy for Stargardt’s Disease

The therapeutic potential of autologous bone marrow-derived stem cells in Stargardt’s disease has been assessed in two clinical trials. A phase 1 trial with an estimated enrollment of 30 patients having retinal degeneration, including those having Stargardt’s disease, started in 2018. Currently, the status of the trial is unknown [132]. Another trial using autologous bone marrow-derived stem cells in patients with retinal degeneration was a single arm, open-label study, titled as Stem Cell Ophthalmology Treatment Study (SCOTS) started in 2012 and completed in 2020 [133]. A subsequent trial Stem Cell Ophthalmology Treatment Study II (SCOTS2) started by the same organization has been started in 2016, with an expected completion in July 2026 [134]. One-year follow-up of patients with Stargardt’s disease from SCOT and SCOT2 trials showed that the disease phenotype improved or remained stable in about 75% of patients (*p* = 0.0004). About 94% of patients had improved vision or remained stable [135].

**Figure 4:**
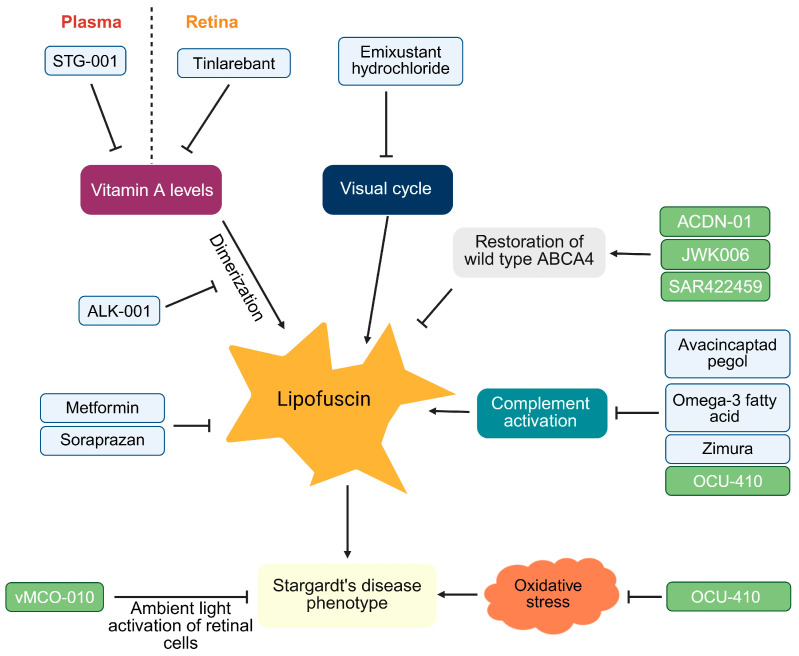
Various therapeutic approaches (small molecules in green and gene therapy in blue) for Stargardt’s disease are shown.

A pictorial summary of various therapeutic approaches that are under clinical development are shown in Figure 4. Despite *ABCA4* mutations being the chief contributor in the etiology of Stargardt’s disease, gene therapies supplementing the wildtype ABCA4 are limited. The relatively large size of ABCA4 mRNA (6.6 kb) limits the use of a AAV-based gene therapy-based approach directly supplementing the wildtype ABCA4 with only one such gene therapy, JWK006, supplementing the wildtype ABCA4 (via an AAV-based vector) under clinical trial (Table 3). Supplementing wildtype ABCA4 via lentiviral or HSV-based vectors that can accommodate larger target gene payloads is limited by intrinsic problems associated with these therapies. Disease-modifying gene therapy approaches, which aim at checking disease progression, provide an alternative to ABCA4 supplementation. As described above, the disease-modifying gene therapy OCU410 that expresses RORA targets two critical aspects of Stargardt’s disease pathology—complement activation and oxidative stress. Preclinical studies in a mouse model of Stargardt’s disease indeed showed a rescue of the disease phenotype. In patients with Stargardt’s disease, OCU410ST treatment resulted in 48% slower lesion growth from baseline and a 10-letter improvement in visual function, as measured by best corrected visual acuity (BCVA), compared to untreated eyes [136]. OCU410 is also being evaluated in patients with geographic atrophy, and the preliminary results from the phase 1/2 trial show a favorable safety and tolerability profile exhibiting slower lesion growth (44%) from the baseline in treated eyes compared to untreated eyes in treated eye versus fellow eyes after single injection [137].

## 2. Conclusions and Future Directions

Stargardt disease was first described in 1909 by Dr. Karl Stargardt, but its genetic basis was identified only in 1997 with the discovery of ABCA4 mutations. Since the initial report of 19 variants, over 1200 pathogenic or likely pathogenic ABCA4 variants have been identified. This extensive genetic heterogeneity, often involving rare variants, complicates genotype–phenotype correlation, further challenged by variable penetrance and geographic variation in allele frequency among healthy individuals.

Only two small molecules have advanced to phase 3 trials: tinlarebant and emixustat hydrochloride. Tinlarebant showed disease stabilization in phase 2 and maintained a favorable safety profile in 1-year interim phase 3 data. Emixustat, in post hoc analysis of the completed phase 3 trial (June 2022), proved to be beneficial only in patients with small baseline lesions; with no further trials, its future remains uncertain.

Gene therapies aimed at restoring wildtype ABCA4 are in early clinical stages. A disease-modifying RORA gene therapy targeting complement activation and oxidative stress shows promise. A light-activated bioengineered opsin also offers a novel approach, with encouraging 24-week results. Stem cell therapy using autologous bone marrow-derived cells has shown positive outcomes at one-year follow-up.

RORA gene therapy, designed to regulate complement activation and oxidative stress, has shown promising results. Preclinical studies of OCU410ST demonstrated safety in cynomolgus monkeys. In the completed Phase I GARDian trial (NCT05956626), treated eyes showed a 48% reduction in atrophic lesion growth and a 2-line (10-letter) BCVA improvement at 6 months. The forthcoming results from ongoing studies will be of significant interest considering encouraging results in both structural and functional assessments. Currently Ocugen is conducting phase 2/3 pivotal confirmatory trial of OCU410ST in patients with Stargardt’s disease.

Despite progress, effective therapies for Stargardt disease remain limited. Given the complexity of the ABCA4 genotype–phenotype relationship, ABCA4-agnostic approaches, such as targeting oxidative stress (RORA gene therapy) and complement inhibition (RORA gene therapy, omega-3 supplementation, and Zimura), may offer broader therapeutic potential.

## Figures and Tables

**Table 1:** Prevalence of various common (prevalence ≥ 5%) mutations across countries.

Variant	Amino Acid Change	Prevalence	Country
3386G>T	R1129L	26% [38]	Spain
		10% [39]	Argentina
5882G>A	G1961E	20% [40]	Italy
		15% [41]	USA
		18% [34]	USA
		13% [37]	Germany
		10% [39]	Argentina
		~8% [38]	Spain
3113C>T	A1038V	9% [37]	Germany
		18% [34]	USA
2894A>G	N965S	10% [42]	China
1622T>C	L541P	9% [37]	Germany
2588G>C	G863A, G863del	7% [37]	Germany
5461-10T>C	T1821Vfs*13, T1821Dfs*6	6% [37]	Germany
		5% [41]	USA

**Table 2:** Fundus autofluorescence (FAF) features are described for various genetic retinopathies.

Disease	FAF Features
Stargardt disease	Central oval area of reduced AF, often surrounded by irregular AF
Best disease	Central round structure with regular or irregular intense AF
Rod-cone dystrophies	Central oval ring-shaped area of increased AF
Early-onset severe retinal dystrophy (with RPE65 mutations)	Complete absence of AF
Leber congenital amaurosis	AF is normal (except in EOSRD with RPE65 mutations)
X-linked retinoschisis	Central radial structures of AF
